# WormJam: A consensus *C. elegans* Metabolic Reconstruction and Metabolomics Community and Workshop Series

**DOI:** 10.1080/21624054.2017.1373939

**Published:** 2017-09-06

**Authors:** Janna Hastings, Abraham Mains, Marta Artal-Sanz, Sven Bergmann, Bart P. Braeckman, Jake Bundy, Filipe Cabreiro, Paul Dobson, Paul Ebert, Jake Hattwell, Hooman Hefzi, Riekelt H. Houtkooper, Rob Jelier, Chintan Joshi, Varun B. Kothamachu, Nathan Lewis, Artur Bastos Lourenço, Yu Nie, Povilas Norvaisas, Juliette Pearce, Cristian Riccio, Nicolas Rodriguez, Toon Santermans, Pasquale Scarcia, Horst Joachim Schirra, Ming Sheng, Reuben Smith, Manusnan Suriyalaksh, Benjamin Towbin, Mary Ann Tuli, Michel van Weeghel, David Weinkove, Aleksandra Zečić, Johannes Zimmermann, Nicolas le Novère, Christoph Kaleta, Michael Witting, Olivia Casanueva

**Affiliations:** a Epigenetics, Babraham Institute, Babraham Research Campus, Cambridge, UK; b Developmental Biology, Andalusian Center for Developmental Biology. Consejo Superior de Investigaciones Científicas/Junta de Andalucia/Universidad Pablo de Olavide, Seville, Spain; c Computational Biology, University of Lausanne, Lausanne, Switzerland; d Department of Biology, University of Gent, Gent, Belgium; e Computational and Systems Medicine, Imperial College London, London, UK; f Structural and Molecular Biology, University College London, London, UK; g School of Computer Science, University of Manchester, Manchester, UK; h School of Biological Sciences, University of Queensland, Queensland, Australia; i Centre for Advanced Imaging, University of Queensland, Queensland, Australia; j Department of Bioengineering, Novo Nordisk Center for Biosustainability at UC San Diego, University of California, San Diego, USA; k Laboratory Genetic Metabolic Diseases, Academic Medical Center, Amsterdam, Netherlands; l Centre of Microbial and Plant Genetics, KU Leuven, Leuven, Belgium; m Department of Pediatrics, Novo Nordisk Center for Biosustainability at UC San Diego, University of California, San Diego, USA; n Signalling, Babraham Institute, Babraham Research Campus, Cambridge, UK; o School of Human and Life Sciences, Canterbury Christ Church University, Canterbury, UK; p Sanger Institute, University of Cambridge, Cambridge, UK; q Dep. Biosciences Biotechnologies Biopharmaceutics, University of Bari, Bari, Italy; r Neurobiology, MRC LMB, Cambridge, UK; s Friedrich Miescher Institute, Basel, Switzerland; t WormBase, Caltech, Pasadena, CA, USA; u Department of Biosciences, Durham University, Durham, UK; v Medical Systems Biology, Christian-Albrechts-University Kiel, Kiel, Germany; w Research Unit Analytical BioGeoChemistry, Helmholtz Zentrum Muenchen, Muenchen, Germany

## Introduction

A GENiE (EU COST action, www.worm-genie.eu) workshop was held at the Babraham Institute in Cambridge, UK on April 19 and 20, 2017, to discuss global challenges around *Caenorhabditis elegans* metabolic reconstructions and metabolomics (http://www.babraham.ac.uk/genie-workshop). This short report describes the outcomes from that workshop, notably the initiation of a global community to collaboratively discover and describe the metabolism of *C. elegans*.

*C. elegans* is a well established workhorse for research in both fundamental biology and medicine, with applications including developmental biology, ageing-associated research and the elucidation of pathomechanisms of diseases.^1^ Powerful molecular as well as genetic tools, ease of cultivation, short generation times and a considerable overlap in its fundamental biology with higher organisms, make *C. elegans* an ideal object of study. In recent years, metabolism is increasingly recognised as an important contributor to *C. elegans* healthspan and lifespan, as well as the basis of adaptation to different environments.^2,3^ Most of the historical efforts to detect changes in metabolism have been based on molecular tools including gene expression and/or reporter strains. Despite its importance for a large number of research applications, efforts to reconstruct the global map of *C. elegans* metabolism only started recently, notably with the publication of two independent constraint-based metabolic models last year^4,5^ (although automated reconstructions from pathway databases were produced before.^6^) Such whole-genome metabolic reconstructions are a key ingredient for a large number of applications including the integration of diverse -omics datasets, investigation of disease-associated changes in metabolism, studying the responses to nutritional interventions, identifying gene-environment interactions, and elucidating inter-organismal interactions on the metabolic level at both physiological and evolutionary timescales.^7^ The reconstruction of *C. elegans* metabolism thus promises to open up the extensive toolbox of constraint-based modelling for *C. elegans* research.

### Creation and expansion of a consensus reconstruction for *C. elegans* metabolism research

During the workshop, ongoing efforts were identified that are merging and reconciling the two published models mentioned above, as well as other unpublished models developed in different labs (http://cai-gsmodel.cai.uq.edu.au),^8^ into consensus reconstructions. The models are partially overlapping and far from complete; many metabolites that can be identified and quantified in *C. elegans* are not represented. Furthermore, the existing models are currently distributed across different databases and web portals. Users wishing to update or extend these models have no provision for merging their changes into any other version, including the original. Ongoing reconciliations between different models are hindered by the lack of agreement on shared standards for the annotation of molecules and reactions.^9^ There is thus an urgent need to agree on standards and a central location for hosting, editing and version management of a master community-wide *C. elegans* reconstruction. This will form the basis for a community effort to expand the consensus model to yet uncovered metabolic pathways relevant in the manifold aspects of *C. elegans* research.

### Metabolomics data and a community driven effort are required to fill the gaps in reconstructions

The documented metabolome of *C. elegans* encompasses around 1,000 metabolites, while the estimated full metabolome may be in the order of >10,000 molecules, based on experiences gathered across different metabolomics labs. Among these molecules, lipids and secondary metabolites are particularly poorly covered. A curation effort based on metabolomics data is under way within WormBase (http://www.wormbase.org/search/molecule/WBMol%3A), but this daunting task requires a dedicated community-driven effort to cover the vast quantity of under-annotated metabolites as well as expert knowledge on the different analytical technologies (NMR and MS) employed to evaluate the quality of the metabolite annotation. Rigorous validation of the experimental approaches in metabolomics and hence curation of data quality is of the utmost importance. Also, even considering only the documented metabolome, there is an overlap of only up to 50% with the current models. A specific example of pathway misrepresentation is that of sphingolipid synthesis, essential for *C. elegans* postembryonic development.^10^ While it is known that in *C. elegans* C17iso-sphingosine is synthesized as sphingoid base from mono-methyl branched chain fatty acid (C15iso),^11^ the published models use palmitic acid instead. Therefore, manual curation and expert knowledge of metabolic pathways is essential to build models that provide accurate predictions.

### The challenges of building metabolic reconstructions for multicellular organisms

Besides the number of metabolites that require further revision, metabolic reconstructions of multicellular organisms are challenging for other reasons. Firstly, tissue-specific differences in metabolic pathways necessitate the long-term goal of generating tissue-specific models. Secondly, recent research suggests that there is a profound impact of the environment on the worm's physiology. In particular, the choice of bacteria as food source, and whether it is provided alive or dead, has a vast impact on metabolism and life history traits.^12,13^ Ongoing efforts in the reconstruction of joint models of *C. elegans* along with its live *E. coli* food source as well as with bacteria that are natural colonizers of the *C. elegans* gut will be required to disentangle bacterial from worm-specific metabolic effects of experimental perturbations. Finally, the peptone-based media that is used to grow bacteria influences both experimental reproducibility and metabolism, calling for a revision of standard worm culture methods to include chemically-defined media.^14^ A consensus metabolic model will allow the prediction of an optimal growth medium, and should therefore be a key ingredient in these efforts.

### WormJam: an open community vision for the future

To achieve these aims, WormJam (short for *Worm Jamboree*) is being established as a platform for a community effort towards reconciliation of existing and expansion of the unified *C. elegans* metabolic reconstruction. With the increasing recognition of metabolism as being of pivotal importance for ageing, development, disease mechanisms as well as evolution, the availability of a community-driven consensus reconstruction of *C. elegans* metabolism will lay the foundation for bringing *C. elegans* to the forefront of metabolism research.

As part of this effort, our objectives for the next 12 months within the WormJam community are:

– Ensuring all existing *C. elegans* metabolic reconstructions and partial consensus reconstructions are freely accessible online.
– Annotation of all molecules that can be identified with confidence in *C. elegans* targeted metabolomics experiments across participating metabolomics labs within the WormBase Molecule resource.
– Establishment of a platform for community curation of the metabolic reconstructions and creation of a consensus *C. elegans* metabolic network. The reconstruction will be tightly integrated with WormBase as a central hub of *C. elegans* data resources.
– Holding annotation jamboree workshops targeting missing *C. elegans* specific pathways and metabolites, the first of which will be held in November 2017 (https://www.helmholtz-muenchen.de/genie-workshop-2017/index.html).
– We are seeking contributors from all aspects of *C. elegans* biology. Please join the discussion at https://groups.google.com/forum/#!forum/wormjam.
